# Developing a Loop-Mediated Isothermal Amplification Assay for the Rapid Detection of Seven Respiratory Viruses including SARS-CoV-2

**DOI:** 10.3390/medicina58091224

**Published:** 2022-09-05

**Authors:** Min-Young Lee, Vu-Minh Phan, Woo-In Lee, Yee-Hyung Kim, Sung-Wook Kang, Tae-Seok Seo

**Affiliations:** 1 Department of Laboratory Medicine, Kyung Hee University Hospital at Gangdong, College of Medicine, Kyung Hee University, Seoul 02447, Korea; 2 Department of Chemical Engineering (BK21 FOUR Integrated Engineering Program), Kyung Hee University, Seoul 17140, Korea; 3 Division of Pulmonary and Critical Care Medicine, Department of Internal Medicine, Kyung Hee University Hospital at Gangdong, College of Medicine, Kyung Hee University, Seoul 02447, Korea

**Keywords:** RT-qLAMP, multiplex, respiratory virus, SARS-CoV-2, RT-qPCR

## Abstract

*Background and Objectives*: The coronavirus disease (COVID-19), caused by the severe acute respiratory syndrome coronavirus 2 (SARS-CoV-2), continues to be a pandemic even in 2022. As the initial symptoms of COVID-19 overlap with those of infections from other respiratory viruses, an accurate and rapid diagnosis of COVID-19 is essential for administering appropriate treatment to patients. Currently, the most widely used method for detecting respiratory viruses is based on real-time polymerase chain reaction (PCR) and includes reverse-transcription real-time quantitative PCR (RT-qPCR). However, RT-qPCR assays require sophisticated facilities and are time-consuming. This study aimed to develop a real-time quantitative loop-mediated isothermal amplification (RT-qLAMP) assay and compare its analytical performance with RT-qPCR. *Materials and Methods*: A total of 315 nasopharyngeal swabs from patients with symptoms of respiratory infections were included in this study. A primary screening of the specimens was performed using RT-qPCR. RNA/DNA from standard strains for respiratory viruses and heat-inactivated preparations of standard strains for SARS-CoV-2 were used to evaluate the accuracy and target specificity of the RT-qLAMP assay. *Results:* We successfully developed an RT-qLAMP assay for seven respiratory viruses: respiratory syncytial virus (RSV) A, RSV B, adenovirus, influenza (Flu) A (H1N1 and H3N2), Flu B, and SARS-CoV-2. RT-qLAMP was performed in a final reaction volume of 9.6 µL. No cross-reactivity was observed. Compared with the RT-PCR results, the sensitivity and specificity of the RT-qLAMP assay were 95.1% and 100%, respectively. The agreement between the two methods was 97.1%. The median amplification time to RT-qLAMP positivity was 22:34 min (range: 6:80–47:98 min). *Conclusions*: The RT-qLAMP assay requires a small number of reagents and samples and is performed with an isothermal reaction. This study established a fast, simple, and sensitive test that can be applied to point-of-care testing devices to facilitate the detection of respiratory viruses, including SARS-CoV-2.

## 1. Introduction

Lower respiratory infections are a major cause of morbidity and mortality worldwide, and in recent years, outbreaks of respiratory infections caused by novel viruses have been frequent. The coronavirus disease 2019 (COVID-19), caused by the severe acute respiratory syndrome coronavirus 2 (SARS-CoV-2), emerged in late 2019, and remains a pandemic even in 2022. The initial symptoms of COVID-19 overlap with those of infections caused by other respiratory viruses, which complicates clinical diagnosis. An accurate and rapid diagnosis of COVID-19 is important to administer appropriate treatment to patients. There is an urgent need to develop rapid detection techniques, as the number of patients with respiratory infections is rapidly increasing owing to the disease, causing an enormous burden on the healthcare system.

Currently, the most widely used method for respiratory virus detection is real-time polymerase chain reaction (PCR) [1]. Reverse transcription-quantitative PCR (RT-qPCR) is the gold standard laboratory test for confirming SARS-CoV-2 infections. However, the RT-qPCR assay relies on sophisticated facilities and well-trained personnel in large hospitals, such as tertiary and university hospitals, and is relatively time-consuming. To curb the spread of respiratory infections exacerbated by large-scale population movements, there is an urgent need for a rapid, point-of-care testing (POCT) device that can provide an early diagnosis and facilitate appropriate treatment, leading to improved patient outcomes.

Isothermal amplification assays, such as loop-mediated isothermal amplification (LAMP), are promising POCT methods with rapidity and simplicity. LAMP amplifies the target gene in isothermal conditions with four to six primers, including four primers selected by combining six parts of a target DNA strand and two loop primers. The LAMP technique does not involve the DNA denaturation stage due to the high strand displacement activity of the Bst DNA polymerase, and can be conducted in isothermal conditions. The assay is highly specific due to the use of several primers and increases the amount of amplified DNA up to a billion copies in less than an hour. The primers combined with Bst DNA polymerase create a dumbbell-like DNA structure. The loop primers, which are complementary to the dumbbell-like DNA, significantly improve the efficiency and sensitivity of the reaction and reduce the time it takes by 50%, producing unique rapid self-priming amplification [2,3,4]. Incorporating LAMP amplification eliminates the need for a sophisticated thermal cycler, and its DNA amplification efficiency beyond the exponential phase significantly shortens the amplification duration [5].

This study aimed to evaluate the analytical performance of LAMP assays for detecting common respiratory viruses in Korea, including SARS-CoV-2, and assessed the correlation between the LAMP assay and RT-qPCR.

## 2. Materials and Methods

### 2.1. Clinical Samples

Nasopharyngeal (NP) swabs were obtained from patients with respiratory infection symptoms at the Kyung Hee University Hospital at Gangdong and Green Cross Laboratories, Republic of Korea. Primary screening for viral pathogens was performed using RT-qPCR. Insufficient sample volumes or samples with mixed infections of more than one virus were excluded from further analysis. RNA extraction was performed using a SEEPREP32 (Seegene, Seoul, Korea), according to the manufacturer’s instructions for the RT-qPCR assays. The left-over samples were frozen at −80 °C until further testing using the reverse transcription-quantitative LAMP (RT-qLAMP) assay. A total of 358 NP specimens were collected, including 228 positive and 130 negative specimens. Forty-two specimens were excluded because of insufficient sample volume or mixed infection with the two viruses.

### 2.2. Design of RT-qLAMP Primers

Primers for RT-qLAMP were designed based on sequences retrieved from the NCBI database (https://www.ncbi.nlm.nih.gov/labs/virus/vssi/#/virus?SeqType_s=Nucleotide, accessed on 1 September 2022). The sequences were aligned using CLC Main Workbench 21 (Qiagen). Primer sets were initially designed using Primer Explorer V5 (Eiken Chemical Co., Ltd., Tokyo, Japan; http://primerexplorer.jp/lampv5e/index.html, accessed on 1 September 2022), then analyzed using NetPrimer (Premier Biosoft, San Francisco, CA, USA; http://www.premierbiosoft.com/NetPrimer/AnalyzePrimerServlet, accessed on 1 September 2022) to verify compatibility. Primer sets included an outer forward primer (F3), an outer backward primer (B3), a forward inner primer (FIP), and a backward inner primer (BIP). To accelerate the reaction when available, loop forward (LF) and loop backward (LB) primers were designed. Detailed information regarding all primer sets is presented in Table 1. All primers were synthesized by Macrogen (Macrogen Inc., Seoul, Korea). Primer solutions for individual assays were prepared and comprised 0.2 µM F3 and B3, 1.6 µM FIP and BIP, and 0.6 µM LF and LB, then stored at −20 °C.

### 2.3. The RT-qLAMP Assay

Individual 10 µL reaction, consisting of 4.4 µL of 2× Mastermix Buffer, 0.2 µL of Enzyme mix composed of both reverse transcriptase and Bst polymerase (Eiken Chemical Co., Ltd., Tokyo, Japan), 0.4 µL of 10X SYBR Green (ThermoFisher Scientific, Waltham, MA, USA), 1 µL of DNase water, and 2.4 µL of a primer solution, was prepared. A 1.6 µL RNA template/clinical specimen was added to each RT-qLAMP reaction, and the amplification was performed using the CFX Connect Detection System (Bio-Rad Laboratories, Hercules, CA, USA) at 64 °C for 50 min.

### 2.4. Evaluation of the Specificity of the RT-qLAMP Assay

We evaluated the accuracy and specificity of the RT-qLAMP assay for detecting respiratory syncytial virus (RSV) subtypes A and B, adenovirus (ADV), influenza (Flu) A (H1N1 and H3N2), Flu B, and SARS-CoV-2. RNA/DNA from rhinovirus (KBPV VR-39), metapneumovirus (KBPV-VR-87), enterovirus (KBPV-VR-58), RSV A (KBPV-VR-73), RSV B (KBPV-VR-42), ADV (KBPV-VR-58), Inf A virus H1N1 (KBPV-KR-76), H3N2 (KBPV-VR-71), and Inf B virus (KBPV-VR-72) was purchased from the Korea Bank for Pathogenic Viruses to evaluate assay target specificity. We also included heat-inactivated preparations of SARS-CoV-2 (ATCC VR-1986) (Koram Biotech Corporation).

### 2.5. The RT-qPCR Assay

Each reaction mixture contained 8 µL of extracted nucleic acid and 17 µL of one-step RT-PCR master mix (5× RP MOM, 5 µL of RNase-free water, 5 µL of 5× real-time one-step buffer, and 2 µL of real-time one-step enzyme) at a final volume of 25 µL. Multiplex RT-qPCR was performed using a CFX96™ real-time PCR System (Bio-Rad Laboratories, Hercules, CA, USA). The mixture was incubated at 50 °C for 20 min for the reverse transcription step, which was followed by denaturation at 95 °C for 15 min and 45 cycles of PCR (10 s at 95 °C, 1 min at 60 °C, 10 s at 72 °C). Fluorescence was detected at two temperatures (60 °C and 72 °C). The results were analyzed automatically using Seegene Viewer V2.0 (Seegene Inc., Seoul, Korea).

### 2.6. Comparison between the RT-qLAMP and RT-PCR

All specimens included in the study were first tested in the clinical laboratory at Kyung Hee University Hospital at Gangdong using the Allplex Respiratory Panels 1/2/3 (Seegene, Seoul, Korea). These assays detect 16 respiratory viruses simultaneously and include Flu A subtyping. In addition, the samples were tested using an Allplex SARS-CoV-2 assay. For a parallel comparison with the RT-qLAMP assay, RT-qPCR, using these panels, was performed again concurrently with the new test to avoid discrepancies in test results due to specimen deterioration during storage.

## 3. Results

### 3.1. Validation RT-qLAMP Assay Using RNA/DNA Template from Standard Strains

**Figure 1:**
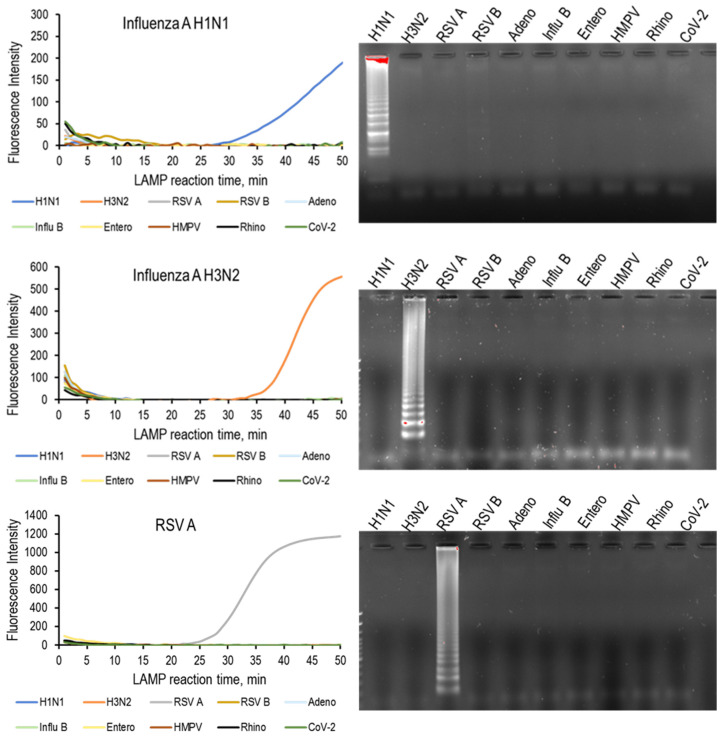

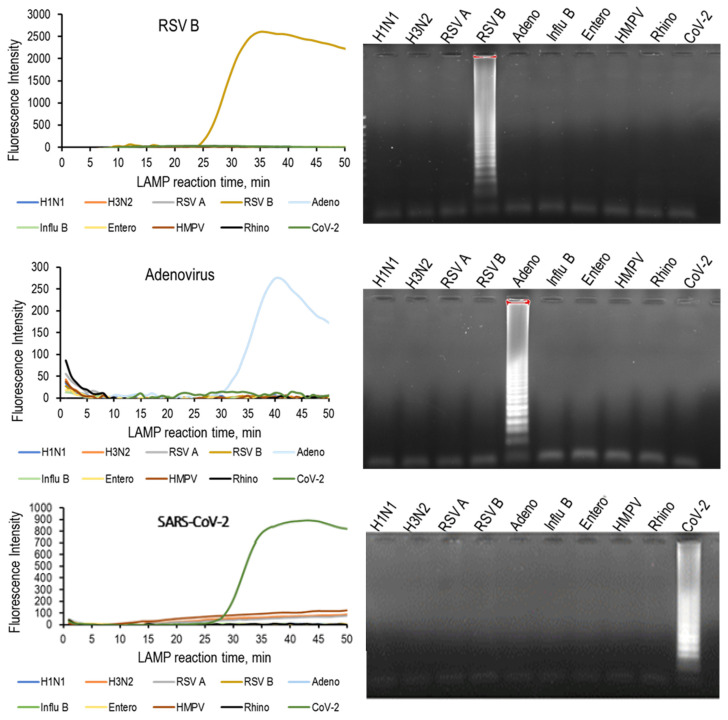
(**Left**) RT-qLAMP results and (**right**) agarose gel electrophoresis of RT-qLAMP products for the corresponding virus with RNA/DNA from standard strains of 10 respiratory viruses. Amplification was observed only for the respective standard strains and no cross-reactivity was observed. RSV: respiratory syncytial virus, HMPV: human metapneumovirus, CoV-2: severe acute respiratory syndrome coronavirus 2.

To assess assay target specificity, RNA/DNA from the 10 standard strains was tested, and an amplification was observed only for the respective standard strains. In addition, for the samples with no amplification, no amplicons were observed by gel electrophoresis (Figure 1). The RT-qLAMP assays developed for RSV A, RSV B, ADV, Flu A (H1N1 and H3N2), Flu B, and SARS-CoV-2 had acceptable specificity against other common respiratory viruses.

### 3.2. Comparison between the RT-qLAMP and RT-qPCR Using Clinical Samples

**Figure 2:**
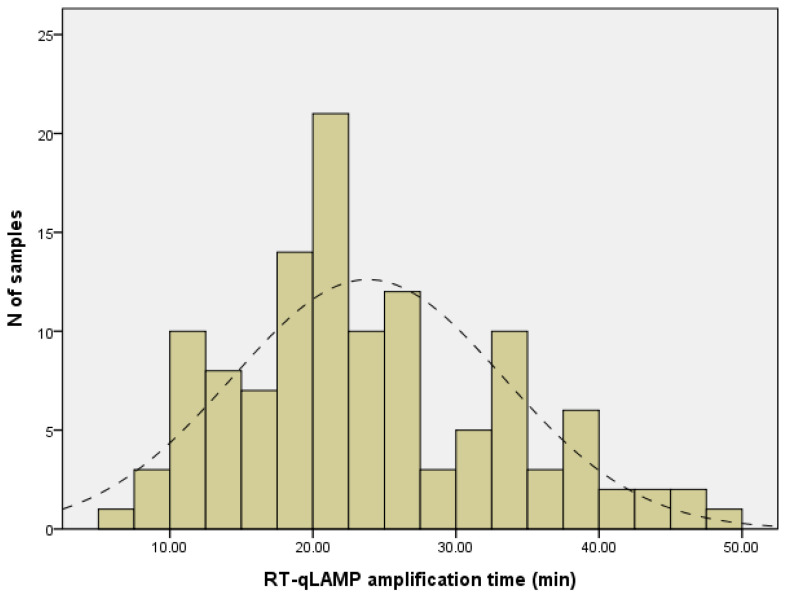
The frequency of amplification time to positivity for RT-qLAMP. The lines of dashes indicate the normal fitted distribution. The mean amplification time to positive results was 22:45 min and all positive test results were obtained within 48 min.

The number of positive and negative NPs finally included based on the primary screening results was 186 and 130, respectively. Among these, RT-qPCR detected a virus in 185 (99.5%), while 176 (95.1%) were positive on RT-qLAMP. Among the 28 samples positive for influenza A-H1N1 with primary RT-qPCR screening, one sample became negative when RT-qPCR was performed again at the same time point as when RT-qLAMP was performed. This is considered to be a matter of sample quality or storage. All 130 NP specimens that were negative by RT-qPCR for 16 respiratory viruses were also negative on the seven RT-qLAMP assays. The detailed results of RT-qLAMP and RT-qPCR for the viruses are shown in Table 2. Compared to RT-qPCR, the sensitivity and specificity of the RT-qLAMP assay were 95.1% and 100%, respectively. The agreement between the two methods was 97.1%. The mean amplification times for the positive samples were as follows: 17:75 min (range, 6:61 to 42:05 min) for ADV positives; 23:21 min (range, 17:53 to 39:11 min) for influenza A-H1N1 positives, 24:41 min (range, 15:34 to 33:93 min) for influenza A-H3N2 positives; 3747 min (range, 25:76 to 47:98 min) for RSV A positives; 19.79 min (range, 17:35 to 21:86 min) for RSV B positives; 25:25 min (range, 15:76 to 41:12 min) for influenza B positives; and 24:55 min (range, 19:23 to 32:31 min) for SARS-CoV-2 positives. The overall median amplification time to RT-qLAMP positivity was 22:45 min (range: 6:61 to 47:98 min) (Figure 2).

## 4. Discussion

Given the rapid spread of respiratory infectious diseases, this study established a fast, simple, and sensitive RT-qLAMP assay that can be applied at point-of-care to facilitate the detection of respiratory viruses, including SARS-CoV-2. Since testing can be performed in local hospital laboratories on demand, extended turnaround times associated with reference laboratory testing can be avoided. Recent publications have reported on assays that were easy to apply to POCT [6,7,8]. However, studies on the development of an assay that can detect viruses that cause respiratory diseases with similar symptoms, including SARS-CoV-2, have rarely been reported.

Our study provides a description of RT-qLAMP test development and an evaluation of test performance in both laboratory and clinical settings. The developed RT-qLAMP assay requires only a small number of reagents and samples (8 µL and 1.6 µL, respectively) in an isothermal reaction at 64 °C. This test facilitates screening for seven respiratory viruses, including SARS-CoV-2, even in a local hospital, with an easy-to-use portable POCT device. For the seven reactions by RT-qLAMP, the cost for the master mix (including enzyme, buffer, SYBR Green 10×) and primer was USD 9.8 and USD 1.41, respectively. Thus, the estimated cost per run of RT-qLAMP is USD 11.21.

Despite its potential as a powerful diagnostic tool, LAMP has not been widely adopted in clinical settings compared to RT-qPCR. One of the major considerations of LAMP performance is assay specificity. False-positive results are common in LAMP reactions, even in the absence of nucleic acid targets [9,10,11]. Therefore, it is crucial to design primer sets with low cross-reactivity to avoid false-positive results. Our results show that the assay had 100% analytical specificity for the identification of all seven viruses and that there was no cross-reaction with other genetically or clinically related reference viruses tested. We attribute the high specificity to the use of six primers instead of four for each gene target. In particular, because influenza viruses have high mutation rates, two sets of primers (12 primers) were used for one gene.

For the optimization of the RT-qLAMP, the test for the limit of detection (LOD) for commercial RT-qPCR was performed to determine the minimum sample volume needed. When positive control materials of 1 × 10^5^~10^6^ copies/µL were used, RT-qPCR showed all positive results until 1 × 10^3^ dilution. Although the LOD of RT-qLAMP has not been directly verified, the LOD of RT-qLAMP is expected to be at least comparable to that of RT-qPCR, considering that the sensitivity was 95% despite the fact that only 20% of the sample volume required for RT-qPCR was used. One previous study also demonstrated that using purified RNA, RT-LAMP could identify samples with 5 to 50 RNA copy numbers, revealing that the accuracy of RT-LAMP was similar to that of RT-PCR [12]. Otherwise, it has been reported that RNA amounts of >100 copies was reliable for performing the antigen test, which is currently the most widely used POCT for detecting SARS-CoV-2 [13].

Our results show that the RT-qLAMP assay for the clinical samples of seven respiratory viruses had a sensitivity and specificity of 95.1% and 100%, respectively. Thus, the sensitivity of our test assay is comparable to the performance of RT-qPCR, the current gold standard. The developed RT-qLAMP showed excellent diagnostic performance in clinical settings. The clinical sensitivity and specificity of the antigen test SARS-CoV-2 are 50% and 97%, respectively [14]. The GeneXpert^®^ molecular diagnostic system, which is widely used because it can be used as a concept of POCT, has both a sensitivity and a specificity of 97%, which is similar to that of the developed RT-qLAMP [15]. Ultimately, a POCT-applied developed RT-qLAMP that can detect multiple respiratory viruses, including SARS-CoV-2, will soon be a clinically useful and promising test device.

The Allplex Respiratory Panel is one of the most commonly used multiplex PCR assays for detecting respiratory viruses in Korea. The test time of the assay is 2 to 5 h, depending on the equipment used [16]. The Allplex SARS-CoV-2 assay also takes approximately 2 h, after nucleic acid extraction, to obtain results [17]. Including the time required for DNA extraction and PCR preparation, both the respiratory panel and SARS-CoV-2 assay take 3 to 6 h to achieve final results. In this study, the reaction time for the RT-qLAMP assay was set at 60 min. Considering the maximum time (47:97 min) for amplification in our study, it can be possible to perform the developed RT-qLAMP assay in approximately 50 min and 30 min for RNA/DNA extraction when examining an unknown specimen.

## 5. Conclusions

We have developed an RT-qLAMP assay that can diagnose seven major respiratory viruses. The RT-qLAMP assay showed similar sensitivity and specificity to RT-qPCR, the reference diagnostic method for SARS-CoV-2. The simplicity, rapid turnaround time, and high sensitivity and specificity of our test make it an attractive and efficient tool for an integrative POCT system, notably in infection control of the mentioned respiratory viruses.

## Figures and Tables

**Table 1:** RT-qLAMP primer sets used in this study.

Virus and Primer Name	Primer Sequences (5′–3′)
ADV	
F3	CTGCTCTCACAGATCACGG
B3	CCCCGCCAAACATCTTGC
FIP	AACGTAGGGGCAGGTGCGG-CAGCATCGGAGGAGTCCA
BIP	CGTCCTATCGAGCCGCACTTT-CCCCAGCCTGTGTTATTGC
LF	CGTCAGTAATGGTCACTCGC
LB	AACATGTCCATCCTTATATCGCC
Flu A/H1N1 (Hemagglutinin gene set 1)	
F3	TGAAGTTACTAATGCTACTGAGCTG
B3	TCCCTCACTTTGGGTCTT
FIP	GACTTTGTTGGTCAGCACTAGTAGTAGATTTTAAAGGGAAAGAAGTCCTCG
BIP	ATCAGAATGCAGATGCATATGTTTTGCTATTTCCGGCTTGAACT
LF	CGATACCCCGTAAGTGGTAG
LB	TTTTGTGGGGACATCAAGATACAG
Flu A/H1N1 (Hemagglutinin gene set 2)	
F3	AGCTAAGAGAGCAATT
B3	TTTCCCTTTATCATTAATGTAGGATTTG
FIP	ACCTTTGTTCGAGTCATGATTGGTCTCAGTGTCATCATTTGAAAGGTTT
BIP	TAACGGCAGCATGTCCTCAGTATGAATTTCCTTTTTTAACTAGCCA
LF	CCATGAACTTGTCTTGGGGAATA
LB	GCTGGAGCAAAAAGCTTCTACA
Flu A/H3N2 (Hemagglutinin gene set 1)	
F3	TGAGCTACATTCTATGTCTGG
B3	GTGAGGACTGTCGCATAT
FIP	CGTTTGGTACTGCATGGTGCTTTTCGCTCAAAAAATTCCTGG
BIP	AGTGAAAACAATCACAAATGACCGCACCTATTGAGGAATTCTGAAC
LF	AGCGTCGCCGTGCTATTGT
LB	TGAAGTTACTAATGCTACTGAGTTGG
Flu A/H3N2 (Hemagglutinin gene set 2)	
F3	CAAGAGAACCTTATGTGTCATG
B3	AACTTGAGCTGGACCATG
FIP	ACTGTGTTATTTGAATGCACGTTGTCAAGTGTTATCAATTTGCCCT
BIP	CGTGATAGGACCCCTTATCGGACTATGCACACTTGCTTGG
LB	ATTGATGAATGAGTTGGGTGTTCCT
RSV A	
F3	GATCTGCAATCGCCAGTG
B3	TCTATCACAGTTTCAATGTTTGA
FIP	TGTGGATAGTAGAGCACTTTTGATTGCATTGCCGTATCCAAGG
BIP	CTAATGGAGTCAGTGTCTTAACCAGGCTTGTTAACAATAGGTAACAACT
LF	GTTCACTTCCCCTTCTAGGTGTA
RSV B	
F3	CACCAGCTGTCAACAACC
B3	GCATTTTTGATCTTGTTCACTT
FIP	TCGTTTCCTCTTCTTGCTTATTGATCAGAAGAGAAGCACCACA
BIP	ATTTCTGGGCTTCTTGTTAGGTGCTCCTTCAAGGTGTAGAACTT
LF	TCTGCAATAGCAAGTGGTATAGCT
Influenza B	
F3	GGACATGAACAACAAAGATGC
B3	GGCAACAAGTTTAGCAACAA
FIP	GGACAATACATTACGCATATCCCTTGATAAAGGAGGAAGTAAACACTCA
BIP	GGAACATTCCTCAAACACCCCAGCCTTCCACTCTGGTCAT
LF	GTCAAACGGAACTTCCCTTCTTTC
LB	GATACAAGTCCTTATCAACTCTGCA
SARS-CoV-2 (orf1ab gene)	
F3	TGCAACTAATAAAGCCACG
B3	CGTCTTTCTGTATGGTAGGATT
FIP	TCTGACTTCAGTACATCAAACGAATAAATACCTGGTGTATACGTTGTC
BIP	GACGCGCAGGGAATGGATAATTCCACTACTTCTTCAGAGACT
LF	TGTTTCAACTGGTTTTGTGCTCCA
LB	TCTTGCCTGCGAAGATCTAAAAC
SARS-CoV-2 (S gene) (S gene)	
F3	CTGACAAAGTTTTCAGATCCTCAG
B3	AGTACCAAAAATCCAGCCTCTT
FIP	TCCCAGAGACATGTATAGCATGGAATCAACTCAGGACTTGTTCTTACC
BIP	TGGTACTAAGAGGTTTGATAACCCTGTTAGACTTCTCAGTGGAAGCA
LF	CCAAGTAACATTGGAAAAGAAA
LB	GTCCTACCATTTAATGATGGTGTTT

ADV: adenovirus, F3: outer forward primer, B3: outer backward primer, FIP: forward inner primer, BIP: backward inner primer, LF: loop forward primer, LB: loop backward primer. RSV: respiratory syncytial virus, SARS-CoV-2: severe acute respiratory syndrome coronavirus 2.

**Table 2:** Comparison of the results between the developed RT-qLAMP and RT-qPCR performed concurrently.

Virus	Multiplex RT-qPCR	RT-qLAMP			Sensitivity, %	Specificity, %
		+	−	Mean Amplification Time, min (Range)		
Flu A/H1N1	+	25	2	23:21 (17:53 to 39:11)	92.6	100
	−	0	288			
Flu A/H3N2	+	14	0	24:41 (15:34 to 33:93)	100	100
	−	0	301			
Flu B	+	3	1	25:25 (15:76 to 41:12)	75	100
	−	0	311			
RSV A	+	26	3	37:47 (25:76 to 47:98)	89.7	100
	−	0	286			
RSV B	+	3	1	19.79 (17:35 to 21:86)	75	100
	−	0	311			
ADV	+	96	1	17:75 (6:61 to 42:05)	99.0	100
	−	0	218			
SARS-CoV-2	+	9	1	24:55 (19:23 to 32:31)	90	100
	−	0	305			
Total	+	176	9	22:34 (6:80 to 47:98)	95.1	100
	−	0	130			

RSV: respiratory syncytial virus, ADV: adenovirus, SARS-CoV-2: severe acute respiratory syndrome coronavirus 2.

## Data Availability

Not applicable.
